# Tryptophan metabolism and small fibre neuropathy: a correlation study

**DOI:** 10.1093/braincomms/fcae103

**Published:** 2024-03-25

**Authors:** Hanae Kushibiki, Hiroki Mizukami, Sho Osonoi, Yuki Takeuchi, Takanori Sasaki, Saori Ogasawara, Kanichiro Wada, Shin Midorikawa, Masaki Ryuzaki, Zhenchao Wang, Takahiro Yamada, Keisuke Yamazaki, Takefusa Tarusawa, Taiyo Tanba, Tatsuya Mikami, Atsushi Matsubara, Yasuyuki Ishibashi, Kenichi Hakamada, Shigeyuki Nakaji

**Affiliations:** Department of Pathology and Molecular Medicine, Biomedical Research Center, Hirosaki University Graduate School of Medicine, Hirosaki, Aomori 036-8562, Japan; Department of Pathology and Molecular Medicine, Biomedical Research Center, Hirosaki University Graduate School of Medicine, Hirosaki, Aomori 036-8562, Japan; Department of Pathology and Molecular Medicine, Biomedical Research Center, Hirosaki University Graduate School of Medicine, Hirosaki, Aomori 036-8562, Japan; Department of Endocrinology and Metabolism, Hirosaki University Graduate School of Medicine, Hirosaki, Aomori 036-8562, Japan; Department of Pathology and Molecular Medicine, Biomedical Research Center, Hirosaki University Graduate School of Medicine, Hirosaki, Aomori 036-8562, Japan; Department of Endocrinology and Metabolism, Hirosaki University Graduate School of Medicine, Hirosaki, Aomori 036-8562, Japan; Department of Pathology and Molecular Medicine, Biomedical Research Center, Hirosaki University Graduate School of Medicine, Hirosaki, Aomori 036-8562, Japan; Department of Pathology and Molecular Medicine, Biomedical Research Center, Hirosaki University Graduate School of Medicine, Hirosaki, Aomori 036-8562, Japan; Department of Orthopaedic Surgery, Hirosaki University Graduate School of Medicine, Hirosaki, Aomori 036-8562, Japan; Department of Pathology and Molecular Medicine, Biomedical Research Center, Hirosaki University Graduate School of Medicine, Hirosaki, Aomori 036-8562, Japan; Department of Otorhinolaryngology-Head and Neck Surgery, Hirosaki University Graduate School of Medicine, Hirosaki, Aomori 036-8562, Japan; Department of Pathology and Molecular Medicine, Biomedical Research Center, Hirosaki University Graduate School of Medicine, Hirosaki, Aomori 036-8562, Japan; Department of Pathology and Molecular Medicine, Biomedical Research Center, Hirosaki University Graduate School of Medicine, Hirosaki, Aomori 036-8562, Japan; Department of Pathology and Molecular Medicine, Biomedical Research Center, Hirosaki University Graduate School of Medicine, Hirosaki, Aomori 036-8562, Japan; Department of Gastroenterological Surgery, Hirosaki University Graduate School of Medicine, Hirosaki, Aomori 036-8562, Japan; Department of Pathology and Molecular Medicine, Biomedical Research Center, Hirosaki University Graduate School of Medicine, Hirosaki, Aomori 036-8562, Japan; Department of Gastroenterological Surgery, Hirosaki University Graduate School of Medicine, Hirosaki, Aomori 036-8562, Japan; Department of Pathology and Molecular Medicine, Biomedical Research Center, Hirosaki University Graduate School of Medicine, Hirosaki, Aomori 036-8562, Japan; Department of Endocrinology and Metabolism, Hirosaki University Graduate School of Medicine, Hirosaki, Aomori 036-8562, Japan; Department of Pathology and Molecular Medicine, Biomedical Research Center, Hirosaki University Graduate School of Medicine, Hirosaki, Aomori 036-8562, Japan; Department of Gastroenterological Surgery, Hirosaki University Graduate School of Medicine, Hirosaki, Aomori 036-8562, Japan; Innovation Center for Health Promotion, Hirosaki University Graduate School of Medicine, Hirosaki, Aomori 036-8562, Japan; Department of Otorhinolaryngology-Head and Neck Surgery, Hirosaki University Graduate School of Medicine, Hirosaki, Aomori 036-8562, Japan; Department of Orthopaedic Surgery, Hirosaki University Graduate School of Medicine, Hirosaki, Aomori 036-8562, Japan; Department of Gastroenterological Surgery, Hirosaki University Graduate School of Medicine, Hirosaki, Aomori 036-8562, Japan; Department of Social Medicine, Hirosaki University Graduate School of Medicine, Hirosaki, Aomori 036-8562, Japan

**Keywords:** pain, diabetic polyneuropathy, small fibre neuropathy, metabolomics, tryptophan

## Abstract

Small nerve fibres located in the epidermis sense pain. Dysfunction of these fibres decreases the pain threshold known as small fibre neuropathy. Diabetes mellitus is accompanied by metabolic changes other than glucose, synergistically eliciting small fibre neuropathy. These findings suggest that various metabolic changes may be involved in small fibre neuropathy. Herein, we explored the correlation between pain sensation and changes in plasma metabolites in healthy Japanese subjects. The pain threshold evaluated from the intraepidermal electrical stimulation was used to quantify pain sensation in a total of 1021 individuals in the 2017 Iwaki Health Promotion Project. Participants with a pain threshold evaluated from the intraepidermal electrical stimulation index <0.20 mA were categorized into the pain threshold evaluated from the intraepidermal electrical stimulation index-low group (*n* = 751); otherwise, they were categorized into the pain threshold evaluated from the intraepidermal electrical stimulation index-high group (*n* = 270). Metabolome analysis of plasma was conducted using capillary electrophoresis time-of-flight mass spectrometry. The metabolite set enrichment analysis revealed that the metabolism of tryptophan was significantly correlated with the pain threshold evaluated from the intraepidermal electrical stimulation index in all participants (*P* < 0.05). The normalized level of tryptophan was significantly decreased in participants with a high pain threshold evaluated from the intraepidermal electrical stimulation index. In addition to univariate linear regression analyses, the correlation between tryptophan concentration and the pain threshold evaluated from the intraepidermal electrical stimulation index remained significant after adjustment for multiple factors (*β* = −0.07615, *P* < 0.05). These findings indicate that specific metabolic changes are involved in the deterioration of pain thresholds. Here, we show that abnormal tryptophan metabolism is significantly correlated with an elevated pain threshold evaluated from the intraepidermal electrical stimulation index in the Japanese population. This correlation provides insight into the pathology and clinical application of small fibre neuropathy.

## Teaser

Kushibiki *et al*. conducted a study to discover new mechanisms and biomarkers of small fibre neuropathy by examining the correlation between pain threshold and serum comprehensive metabolites in >1000 healthy general population. The results showed that serum tryptophan level and metabolism significantly correlated with worsened pain threshold.

## Graphical Abstract

**Graphical Abstract:**
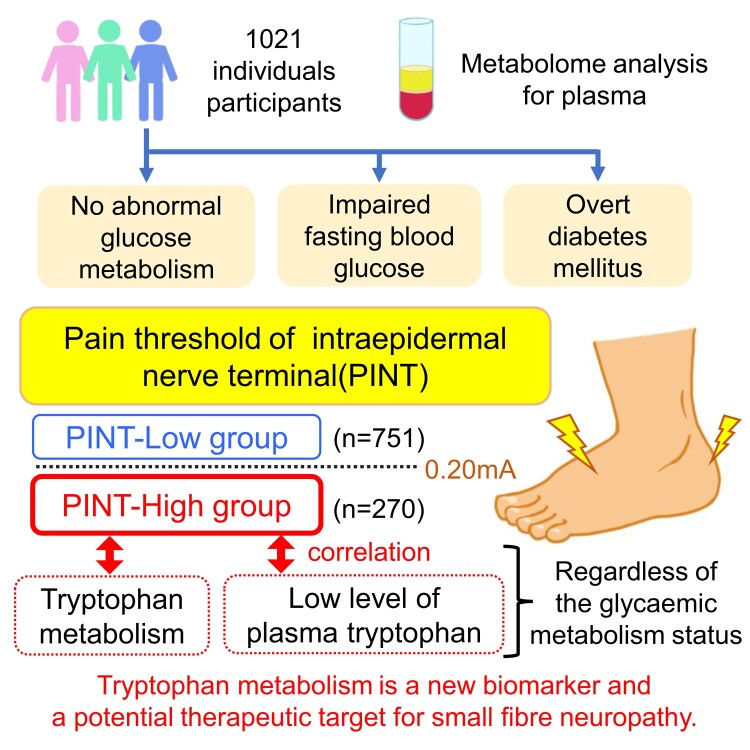

## Introduction

Peripheral nerve fibres can be classified according to their size, which correlates with the degree of myelination.^1^ Large nerve fibres are heavily myelinated and include A-α fibres, which mediate motor strength, and A-β fibres, which mediate vibratory and touch sensations. Medium-sized fibres, known as A-γ fibres, are also myelinated and carry information to muscle spindles. Small fibres include myelinated A-δ fibres and unmyelinated C fibres, which innervate skin (somatic fibres) and involuntary muscles, including cardiac and smooth muscles (autonomic fibres). Together, they mediate pain, thermal sensation and autonomic function.

Since small nerve fibres escape electrophysiological tests for nerve conduction velocities, a diagnostic ‘gold standard’ for small fibre dysfunction has not been established.^2^ The evaluation of small nerve fibre function is currently based on the typical pain history, pathological analysis, such as finding reduced distal intraepidermal nerve fibre density in skin punch biopsies and noninvasive corneal confocal microscopy, and nonpathological analysis, such as finding elevated temperature thresholds in the quantitative sensory testing without signs of large fibre impairment or sudoscan.^3-9^ In addition, the pain threshold reflects the function of small nerve fibres, because thinly myelinated A-δ fibres and unmyelinated C fibres sense temperature changes and pain in the epidermis.^10^ The pain threshold can be evaluated as the index of the pain threshold from intraepidermal electrical stimulation (PINT) by employing an electrode for intraepidermal electrical stimulation (IES).^11-17^ There was a significant increase in the PINT in patients with small fibre dysfunction, such as diabetic polyneuropathy (DPN), compared with that in patients without DPN.^12,13^

Disorders of these nerve fibres manifest as small fibre neuropathy (SFN).^18^ These nerve fibres are generally affected by the prediabetic state of DPN and result in spontaneous pain or loss of pain sensation, which are the symptoms of SFN.^19,20^ We have clarified that SFN can be associated with polygenic factors, including factors associated with diabetes, as well as nondiabetic factors, such as changes in the gut microbiota.^14-17^

Interestingly, in addition to glucose metabolism and dyslipidaemia, disturbances in amino acid metabolism, such as decreased baseline levels of asparagine, glutamine and serine, are known to be correlated with DPN, including diabetic autonomic neuropathy.^21-23^ Since it is recognized that the pathogenesis of DPN is caused by a complex condition in which metabolic failure of multiple nutrients is involved, the pathogenesis of SFN may also involve multiple metabolic deficiencies.

The recent development of high-throughput metabolomic technologies certainly poses many unprecedented opportunities for studies of complex chronic diseases, including peripheral polyneuropathy.^24^ Previous reports applying metabolomics have shown that glucosamine, which is metabolized via the hexosamine pathway, accumulates in the sciatic nerves and that the level of serine is low in experimental DPN.^23,25^ Blood can also be an ideal sample source for detecting indirect metabolic changes in individuals with peripheral neuropathy, including neuropathic pain and DPN.^26-30^ However, to date, no study has reported the association between pain threshold and changes in blood metabolites.

Therefore, our study is intended to explore the significant changes in blood metabolites and metabolic pathways reflecting the mechanism of the deterioration of the pain threshold in a healthy Japanese population in units of 1000s.

## Materials and methods

### Ethical approval and patient consent

This study was performed in accordance with the ethical standards of the Declaration of Helsinki and approved by the ethics committee of the Hirosaki University Medical Ethics Committee (# 2021-030). All patients provided written informed consent for this study.

### Demographic characteristics of the study participants

The medical data of the volunteers were obtained from the Iwaki Health Promotion Project (Iwaki Study), a health promotion study of Japanese citizens over 20 years of age.^31^ In this project, a health evaluation was conducted annually for participants living in the Iwaki area, a suburban area of Hirosaki in the Aomori Prefecture of northern Japan.^14-17,31^ Associations between clinical measurements and PINT indices were examined using the data from the 2017 Iwaki Study.

### Clinical profile

Blood samples were collected in the morning from peripheral veins with the individual in the supine position after a period of fasting for >10 hours. The following clinical data were recorded: height; body weight; body mass index (BMI); percent body fat (Fat); waist circumference; fasting blood glucose (FBG); glycated haemoglobin A1C (HbA1c); fasting immunoreactive insulin (F-IRI); homeostasis model assessment for β cell function (HOMA-β) and insulin resistance (HOMA-IR); blood urea nitrogen (BUN); creatinine (Cr); systolic blood pressure (sBP); diastolic BP (dBP); total cholesterol (Tc), and triglyceride (Tg) levels; high-density lipoprotein cholesterol (HDL-c); low-density lipoprotein cholesterol (LDL-c); interleukin-6 (IL-6); high-sensitivity C-reactive protein (Hs-CRP); and pentosidine. Urine levels of 8-hydroxy-2′-deoxyguanosine (8-OHdG) were also measured. The adipose tissue volume was measured via the bioelectricity impedance method using a Tanita MC-190 body composition analyser (Tanita Corp., Tokyo, Japan). Diabetes was diagnosed according to the 2010 Japan Diabetes Society criteria [impaired fasting glucose (IFG): FBG levels 110–125 mg/dL; diabetes: FBG levels ≥ 126 mg/dL or HbA1c levels ≥ 6.5%].^32^ Those on medication for diabetes with a normal blood glucose level were also defined as having diabetes. HbA1c (%) was expressed as the National Glycohemoglobin Standardization Program value. None of the patients were diagnosed with type 1 diabetes or inherited diseases that affected HbA1c values. Hypertension was defined as a blood pressure ≥ 140/90 mmHg or a history of treatment for hypertension. Dyslipidaemia was defined as Tc ≥ 220 mg/dL, Tg ≥ 150 mg/dL or history of treatment for hyperlipidaemia. Alcohol intake (current or nondrinker), smoking habits (current or nonsmoker) and subjective neuropathic foot symptoms (pricking, burning and aching pains) were determined via questionnaire. The Achilles tendon reflex (ATR) was scored based on two titre systems: scores 0, areflexia/hyporeflexia, and 1, normal/hyperreflexia.^14^

### PINT measurement

The PINT procedure was performed according to the same methodology that was employed in previous studies.^12-17^ For the IES method, a disposable concentric bipolar needle electrode (NM-983 W; Nihon Kohden Corp., Tokyo, Japan) was connected to a specific stimulator for cutaneous A-δ and C fibres as previously described (PNS-7000; Nihon Kohden).^12-17^ The IES electrode was basically placed on the skin of the centre instep in all participants. In addition, the skin over the extensor digitorum brevis was further evaluated in approximately half of the randomly selected participants.^14-17^ Because this stimulation evokes a local pricking sensation, the thick keratinized layer of the skin can interrupt the electronic stimulation. In such cases, a location with less keratinization was selected on the same foot. The participants pushed the button as quickly as possible if they could feel a sensation. The electrical stimulation intensity started from 0.4 mA and decreased stepwise by 0.05 mA until the participants could no longer sense a pricking sensation. The PINT index was defined as the minimum current threshold that participants could sense in more than two trials. Therefore, the PINT can objectively quantitate the degree of hypoalgesia. The PINT measurements were performed by a total of 20 well-trained staff members. For all subjects, the median PINT index was 0.10 mA, and the average PINT index was 0.15 ± 0.14 mA, with a 95% confidence interval ranging from 0.14 to 0.16. The 95th percentile was 0.16 mA. Therefore, subjects with a PINT index <0.20 mA were classified as PINT-Low subjects, otherwise PINT-High subjects in this study.^14^

### Blood sampling and metabolite extraction

After centrifugation of the blood samples, the collected plasma was separately stored at −80°C. Metabolite extraction and metabolome analysis were conducted at Human Metabolome Technologies (HMT, Tsuruoka, Yamagata, Japan), as described elsewhere.^33^ Briefly, 50 μL of plasma was added to 450 μL of methanol containing internal standards (Solution ID: H3304-1002, HMT, Inc., Tsuruoka, Japan) at 0°C to inactivate enzymes. The extract solution was thoroughly mixed with 500 μL of chloroform and 200 μL of Milli-Q water and centrifuged at 2300*×g* and 4°C for 5 min. Then, 350 μL of the upper aqueous layer was centrifugally filtered through a Millipore 5-kDa cut-off filter to remove the proteins. The filtrate was subsequently centrifuged. The pellet was resuspended in 50 μL of Milli-Q water and then used for metabolome analysis at the HMT.

### Metabolome analysis

Metabolome analysis was conducted with the Basic Scan package of the HMT using capillary electrophoresis time-of-flight mass spectrometry based on the methods described previously.^25,33-39^ VANTED software was used to plot detected metabolites on metabolic pathway maps.^39^

### Statistical analysis

In this study, no statistical sample size calculations were conducted. However, the sample size was determined based on a previous similar study.^33^ Statistical analyses of the clinical data were performed using JMP ver. 12.1 (SAS Institute, Cary, NC) and R software (R Foundation for Statistical Computing, version R-3.4.3). The values of clinical measures are expressed as the means ± standard deviations. The normality of the data was evaluated by the Shapiro–Wilk normality test and Kolmogorov–Smirnov normality test. If the factors were normally distributed, *Z*-score normalization was performed. Because the PINT indices were not normally distributed, *Z*-score normalization was applied.

For the assessment of the PINT index and clinical parameters, one-way analysis of variance with *post hoc* tests, *χ*^2^ tests, Wilcoxon rank sum test, linear regression analyses and multiple logistic regression analysis was performed. One-way analysis of variance with *post hoc* tests, *χ*^2^ tests and Wilcoxon rank sum tests was performed to determine the statistical significance of the difference in values between two groups (parametric or nonparametric) and case–control associations among groups (nonparametric), respectively. Linear regression analyses were assessed for correlations between PINT index and clinical parameters. Multiple logistic regression analysis was used for further assessment of the multivariate correlations. Values were adjusted for factors associated with the PINT index using univariate regression analysis and accounting for potentially cofounding variables for SFN, as reported in a previous study.^14-17^

For the assessment of metabolite data, Welch’s *t*-test, principal coordinate analysis (PCA) and metabolite set enrichment analysis (MSEA) were performed with the Kyoto Encyclopedia of Genes and Genomes database. The metabolite data were filtered and normalized using default settings in MetaboAnalystR (3.2.0; Supplementary Fig. 1).^40^ In brief, the following preprocessing steps were applied: metabolites with a total count of 100 or more and a value of zero for each metabolite were removed and further filtered based on the interquartile range. Zero values were then replaced with half of the smallest positive value in the original data set. Next, the data set was normalized using the median value. Finally, the normalized data were log transformed to the bottom 10.

With respect to the PINT cut-off of 0.2 mA, the data set excluding diabetes included 37 metabolites and 889 individuals (662 controls and 227 cases), and the data set with diabetes included 37 metabolites and 1021 individuals (751 controls and 270 cases). MSEA with the Kyoto Encyclopedia of Genes and Genomes database was performed using MetaboAnalystR (3.2.0) to explore the pathways related to fluctuations in the PINT index. Welch’s *t*-test for the statistical significance of the difference in values between two groups (parametric) and PCA for visualization of the beta diversity for metabolites were performed using R. A value of *P* < 0.05 was regarded as statistically significant.

## Results

### Subject demographics

**Figure 1:**
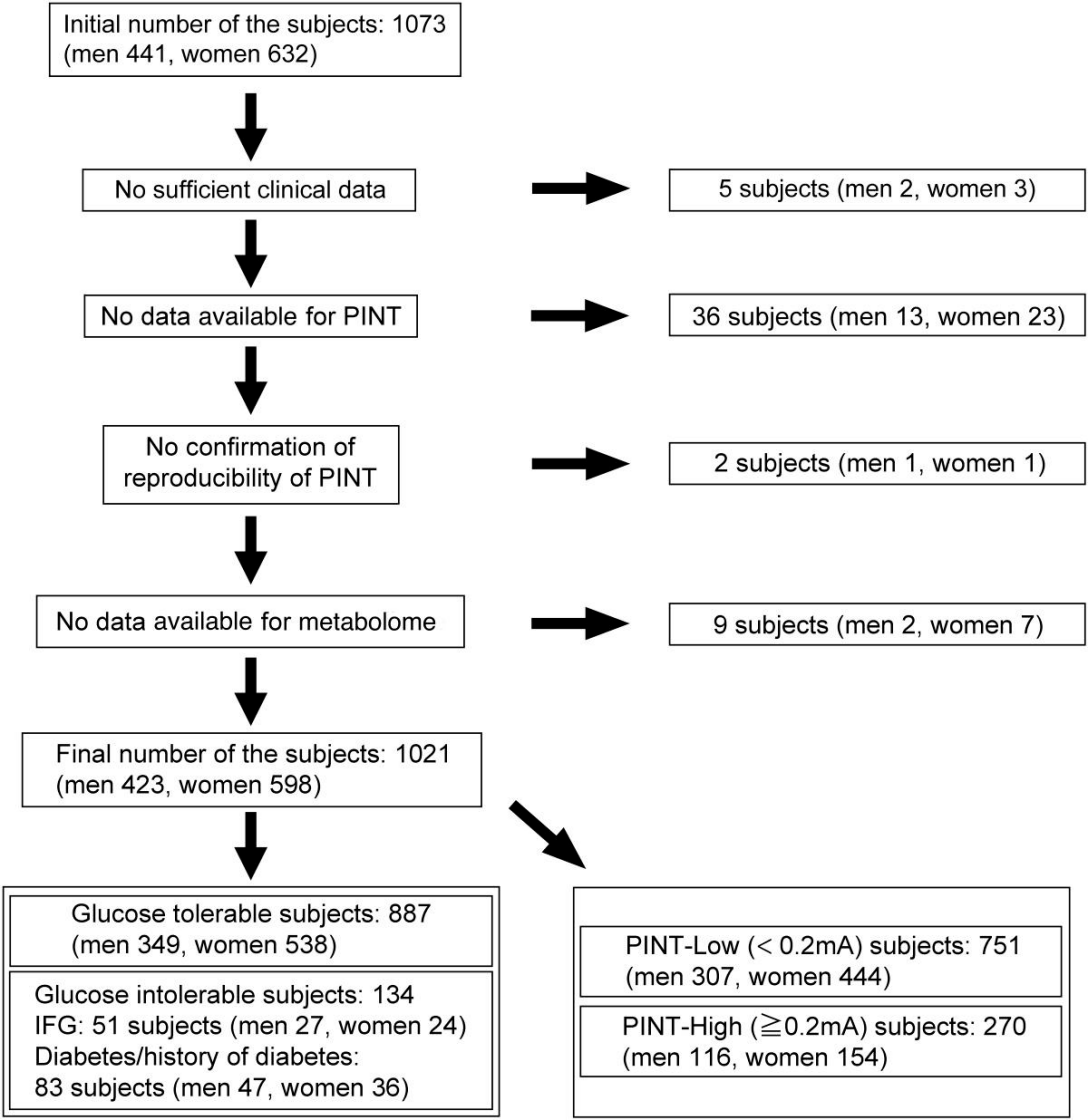
**Flowchart of subject selection.** Of the 1073 volunteers included in the Iwaki Study 2017, 887 normoglycaemic participants (349 males, 538 females), 51 patients with IFG (27 males, 24 females) and 83 patients with type 2 diabetes (47 males, 36 females) were ultimately examined in this study. The participants were also categorized as 751 PINT-Low subjects (307 males, 444 females) or 270 PINT-High subjects (116 males, 154 females) based on the PINT index. PINT, pain threshold from intraepidermal electrical stimulation; IFG, impaired fasting glucose.

Out of 1073 volunteers from the Iwaki Study 2017, 1021 subjects (423 men, 598 women), including overt diabetic subjects, were ultimately examined in this study (Fig. 1). The clinical profiles of the participants based on sex differences are shown in Table 1. The mean age was 54.09 ± 15.50 years for men and 54.55 ± 15.15 years for women. Although FBG was significantly greater in men than in women (*P* < 0.001), HbA1c was comparable between men and women. The parameters reflecting inflammatory status, such as IL-6 and Hs-CRP levels, were comparable between men and women. The PINT indices were also comparable between men and women (0.16 ± 0.14 mA versus 0.15 ± 0.14 mA).

**Table 1:** Clinical profiles of examined subjects divided by sex

	Men	Women	*P*
*n*	423	598	
Age (years)	54.09 ± 15.50	54.55 ± 15.15	0.64
Height (cm)	168.72 ± 6.62	155.86 ± 6.25	<0.0001
Body weight (kg)	68.17 ± 11.23	54.37 ± 9.32	<0.0001
BMI (kg/m^2^)	23.86 ± 3.57	22.37 ± 3.86	<0.0001
Fat (%)	20.61 ± 5.95	29.95 ± 7.48	<0.0001
Waist circumference (cm)	83.98 ± 9.03	74.3 ± 9.69	<0.0001
FBG (mg/dL)	98.68 ± 19.43	92.27 ± 13.15	<0.0001
HbA1c (%)	5.73 ± 0.74	5.67 ± 0.55	0.16
F-IRI (μU/mL)	5.80 ± 8.03	5.31 ± 3.23	0.17
HOMA-β	58.55 ± 41.41	68.95 ± 32.60	<0.0001
HOMA-IR	1.47 ± 1.96	1.25 ± 0.89	<0.05
BUN (mg/dL)	15.38 ± 4.26	14.06 ± 3.93	<0.001
Cr (mg/dL)	0.86 ± 0.38	0.63 ± 0.22	<0.001
sBP (mmHg)	126.29 ± 18.03	120.91 ± 17.59	<0.0001
dBP (mmHg)	73.98 ± 11.77	69.81 ± 11.10	<0.0001
Tc (mg/dL)	203.54 ± 32.33	209.64 ± 35.26	<0.01
Tg (mg/dL)	123.96 ± 90.84	81.10 ± 43.63	<0.0001
HDL-c (mg/dL)	59.26 ± 16.61	70.03 ± 16.37	<0.0001
LDL-c (mg/dL)	114.24 ± 27.41	116.71 ± 29.29	0.18
IL-6 (pg/mL)	1.66 ± 3.56	1.41 ± 4.37	0.33
Hs-CRP (mg/dL)	0.07 ± 0.09	0.06 ± 0.09	0.12
Pentosidine (pmol/mL)	29.78 ± 19.10	31.16 ± 19.53	0.26
Urine 8-OHdG (ng/mg·Cr)	8.64 ± 3.61	9.36 ± 4.58	<0.01
Hypertension: *n* (%)	285/423 (32.62)	138/600 (25.83)	<0.0001
Dyslipidaemia: *n* (%)	56/423 (13.24)	86/600 (14.33)	0.65
Alcohol habit: *n* (%)	294/423 (69.50)	193/600 (32.17)	<0.0001
Smoking habit: *n* (%)	109/423 (25.77)	48/600 (8.0)	<0.0001
Decreased ATR: *n* (%)	102/421 (24.23)	93/593 (15.68)	<0.001
Subjective symptom: *n* (%)	8/423 (1.89)	11/600 (1.83)	>0.9999
PINT (mA)	0.16 ± 0.14	0.15 ± 0.14	0.41

BMI, body mass index; Fat, percent body fat; FBG, fasting plasma glucose; F-IRI, fasting immunoreactive insulin; HOMA-β, homeostatic model assessment β cell function; HOMA-IR, homeostatic model assessment insulin resistance; sBP, systolic blood pressure; dBP, diastolic blood pressure; Tc, total cholesterol; Tg, triglyceride; HDL-c, high-density lipoprotein cholesterol; LDL-c, low-density lipoprotein cholesterol; IL-6, interleukin-6; Hs-CRP, high-sensitivity C-reactive protein; 8-OHdG, 8-hydroxy-2′-deoxyguanosine; ATR, Achilles tendon reflex; PINT, pain threshold of intraepidermal electrical stimulation.

The participants were also divided into 887 glucose-tolerant subjects (349 men, 538 women) and 134 glucose-intolerant subjects (74 men, 60 women). Glucose-intolerant subjects were further divided into an IFG group of 51 individuals (27 men, 24 women) and an overt diabetes group of 83 individuals (47 men, 36 women) according to the criteria of the Japanese Diabetes Society.^32^ The clinical profiles of the participants based on glycaemic condition are shown in Supplementary Table 1. The mean age was higher in abnormal glycaemic subjects than in control subjects (52.87 ± 15.22 years for control group, 65.94 ± 10.30 years for IFG group and 63.17 ± 12.51 years for diabetic group, *P* < 0.0001 versus control, respectively). FBG, HbA1c and HOMA-IR levels increased in a stepwise manner according to the deterioration of the glycaemic condition. Hs-CRP and pentosidine levels were significantly higher in the diabetic group than in the control and IFG groups (*P* < 0.001 versus control and *P* < 0.05 versus IFG). These findings reflect both inflammatory properties and high glucose levels in diabetic patients. Although the PINT index of the IFG was significantly greater than that of control group (0.21 ± 0.21 mA versus 0.15 ± 0.13 mA), the PINT indices were comparable between the control and diabetic groups (0.15 ± 0.13 mA versus 0.17 ± 0.15 mA). These differences might be ascribed to the therapeutic intervention in the diabetic groups.

The participants were divided into the PINT-Low group (<0.2 mA) and the PINT-High group (≥0.2 mA). The clinical profiles of the participants based on the PINT index are shown in Supplementary Table 2. The mean age was 53.45 ± 15.17 years for PINT-Low and 56.88 ± 15.37 years for PINT-High (*P* < 0.001). FBG and HbA1c levels were significantly greater in the PINT-High group than in the PINT-Low group (*P* < 0.01). IL-6, Hs-CRP and pentosidine levels were comparable between the two groups. PINT index was significantly greater in the PINT-High group than in the PINT-Low group (0.34 ± 0.15 mA versus 0.09 ± 0.04 mA, *P* < 0.0001).

### Metabolites and peripheral pain sensation

**Figure 2:**
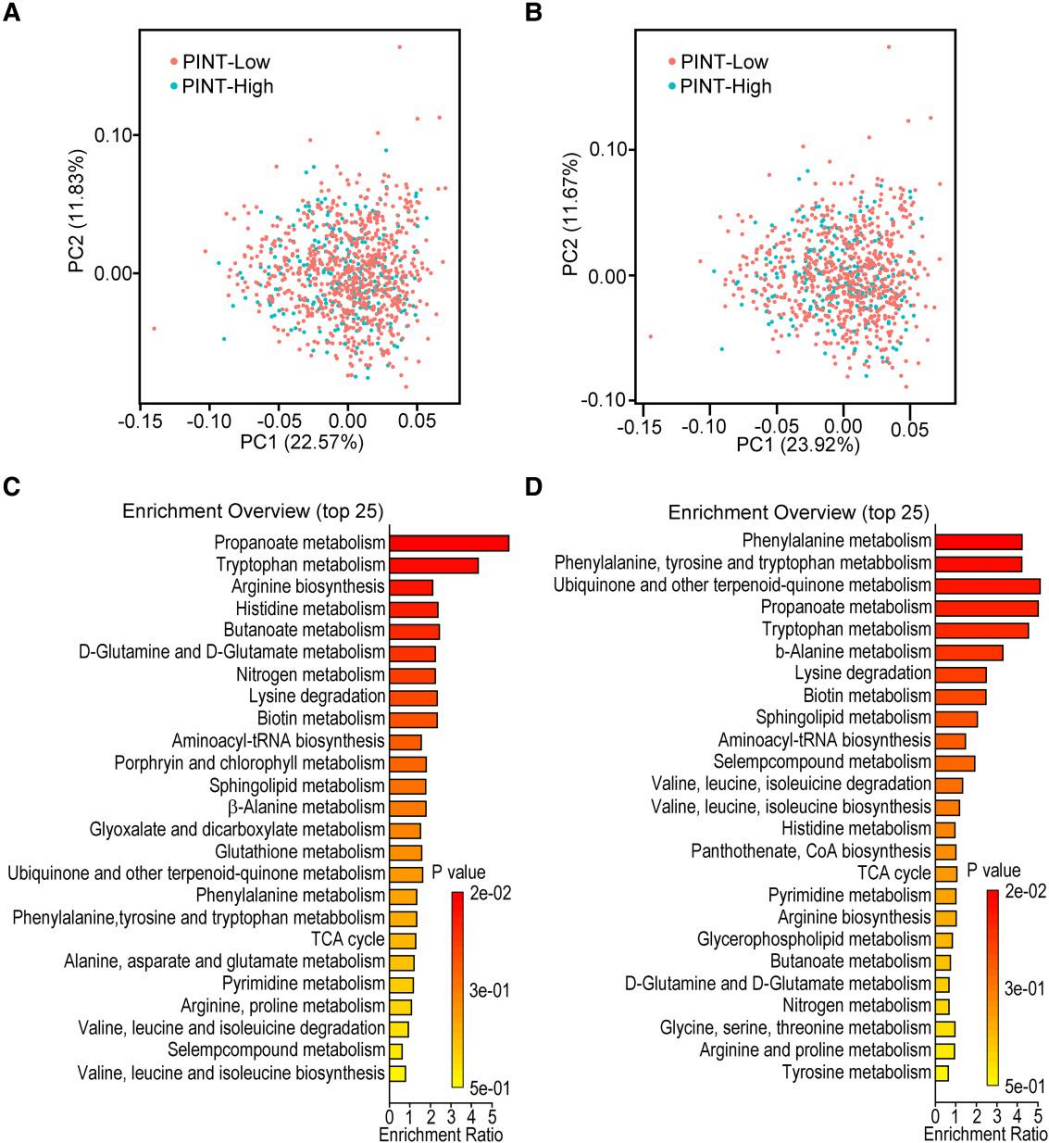
**Variety of plasma metabolite abundance.** PCA between PINT-Low (<0.20 mA) and PINT-High (≥0.20 mA) participants is shown for all subjects (**A**) and for the subjects without abnormal glucose levels (**B**). The top 25 enrichment pathways evaluated by MSEA for all subjects (**C**) and for the subjects without abnormal glucose levels (**D**) are shown. PINT, pain threshold from intraepidermal electrical stimulation; TCA, tricarboxylic acid cycle.

PCA revealed no significant differences in metabolites between the PINT-Low and PINT-High participants, including subjects with diabetes and IFG (Fig. 2A). After excluding the individuals with diabetes and IFG, a significant difference was not observed (Fig. 2B). These findings suggest that all metabolic states of the PINT-High participants were minimally influenced by changes in pain thresholds. On the other hand, the five pathways revealed by MSEA analysis included propanoate metabolism (*P* < 0.05), tryptophan metabolism (*P* < 0.05), arginine biosynthesis (*P* = 0.10), histidine metabolism (*P* = 0.12) and butanoate metabolism (*P* = 0.12), and the top two pathways were significantly correlated with the PINT index in all subjects (Fig. 2C). Excluding IFG and diabetic subjects, propanoate metabolism (*P* < 0.05) and tryptophan metabolism (*P* < 0.05) remained in the top five pathways by MSEA analysis (Fig. 2D). Thus, these two pathways are thought to be specifically related to pain thresholds regardless of blood glucose metabolism.

### Metabolite change in the serum of the subjects

After standardization and filtering, metabolite levels were normally distributed, and 37 metabolites were identified for the subsequent analysis (Supplementary Fig. 1; Table 2). In the comparison between the PINT-Low and PINT-High index groups, 2-hydroxybutyric acid (−0.12 ± 0.17 versus −0.09 ± 0.15, *P* < 0.01) and cis-aconitic acid (−0.71 ± 0.15 versus −0.69 ± 0.12, *P* < 0.05) were identified as metabolites with significantly increased levels in patients with a high PINT index. Conversely, tryptophan was identified as the metabolite that was significantly decreased in subjects with a high PINT index (−0.07 ± 0.08 versus −0.08 ± 0.08, *P* < 0.05). Excluding the subjects manifesting IFG and diabetes, 2-hydroxybutyric acid (−0.12 ± 0.17 versus −0.09 ± 0.15, *P* < 0.05) and tyrosine (0.02 ± 0.07 versus 0.03 ± 0.06, *P* < 0.05) were significantly increased, while tryptophan was similarly decreased (−0.06 ± 0.08 versus −0.07 ± 0.08, *P* < 0.05; Table 3). Combined with the results of the MSEA analysis, tryptophan metabolism is speculated to be most strongly correlated with the pain threshold, regardless of glucose metabolic status.

**Table 2:** Metabolite changes in the serum of all subjects

Metabolite	PINT-Low	PINT-High	*P*
2-Hydroxybutyric acid	−0.12 ± 0.17	−0.09 ± 0.15	<0.01
2-Oxoisovaleric acid	−0.58 ± 0.09	−0.589 ± 0.08	0.55
3-Hydroxybutyric acid	0.10 ± 0.34	0.14 ± 0.33	0.13
Alanine	0.79 ± 0.09	0.78 ± 0.09	0.42
Arginine	0.19 ± 0.10	0.17 ± 0.09	0.06
Asparagine	−0.11 ± 0.08	−0.12 ± 0.08	0.23
Betaine	0.06 ± 0.10	0.05 ± 0.10	0.06
Choline	−0.53 ± 0.10	−0.53 ± 0.11	0.66
cis-Aconitic acid	−0.71 ± 0.15	−0.69 ± 0.12	<0.05
Citric acid	0.54 ± 0.11	0.54 ± 0.11	0.61
Citrulline	−0.25 ± 0.11	−0.26 ± 0.11	0.15
Creatine	−0.17 ± 0.23	−0.16 ± 0.21	0.51
Creatinine	−0.03 ± 0.10	−0.03 ± 0.10	0.94
Glutamic acid	−0.18 ± 0.21	−0.16 ± 0.22	0.13
Glutamine	1.11 ± 0.07	1.11 ± 0.07	0.54
Glycine	0.70 ± 0.13	0.70 ± 0.14	0.66
Histidine	0.19 ± 0.07	0.18 ± 0.08	0.23
Hydroxyproline	−0.82 ± 0.16	−0.81 ± 0.17	0.32
Hypoxanthine	−1.51 ± 0.22	−1.51 ± 0.22	0.78
Isoleucine	0.01 ± 0.08	0.01 ± 0.08	0.15
L-Proline	0.40 ± 0.11	0.40 ± 0.11	0.54
Lactic acid	1.42 ± 0.13	1.42 ± 0.13	0.74
Leucine	0.29 ± 0.06	0.29 ± 0.06	0.83
Lysine	0.63 ± 0.07	0.62 ± 0.07	0.12
Malic acid	−0.91 ± 0.22	−0.90 ± 0.21	0.58
Methionine	−0.56 ± 0.10	−0.56 ± 0.11	0.99
*N*,*N*-Dimethylglycine	−1.13 ± 0.14	−1.11 ± 0.13	0.17
Ornithine	0.01 ± 0.09	0.01 ± 0.11	0.77
Phenylalanine	−0.03 ± 0.05	−0.02 ± 0.06	0.37
Pyruvic acid	0.40 ± 0.19	0.40 ± 0.15	0.83
Sarcosine	−1.37 ± 0.14	−1.37 ± 0.13	0.57
Serine	0.33 ± 0.11	0.32 ± 0.10	0.18
Threonine	0.37 ± 0.11	0.36 ± 0.07	0.46
Tryptophan	−0.07 ± 0.08	−0.08 ± 0.08	<0.05
Tyrosine	0.02 ± 0.07	0.02 ± 0.07	0.21
Uridine	−0.67 ± 0.09	−0.66 ± 0.09	0.20
Valine	0.61 ± 0.05	0.60 ± 0.06	0.36

PINT, pain threshold of intraepidermal electrical stimulation.

**Table 3:** Metabolite changes in the serum of subjects without dysglycaemia

Metabolite	PINT-Low	PINT-High	*P*
2-Hydroxybutyric acid	−0.12 ± 0.17	−0.09 ± 0.15	<0.05
2-Oxoisovaleric acid	−0.58 ± 0.09	−0.58 ± 0.08	0.29
3-Hydroxybutyric acid	0.11 ± 0.35	0.14 ± 0.33	0.24
Alanine	0.78 ± 0.09	0.77 ± 0.08	0.14
Arginine	0.19 ± 0.11	0.18 ± 0.09	0.08
Asparagine	−0.10 ± 0.08	−0.11 ± 0.07	0.10
Betaine	0.06 ± 0.10	0.05 ± 0.10	0.15
Choline	−0.53 ± 0.10	−0.54 ± 0.11	0.39
cis-Aconitic acid	−0.72 ± 0.15	−0.70 ± 0.12	0.06
Citric acid	0.54 ± 0.12	0.55 ± 0.11	0.42
Citrulline	−0.25 ± 0.11	−0.26 ± 0.10	0.20
Creatine	−0.16 ± 0.22	−0.15 ± 0.20	0.33
Creatinine	−0.03 ± 0.09	−0.04 ± 0.08	0.63
Glutamic acid	−0.19 ± 0.21	−0.18 ± 0.22	0.40
Glutamine	1.11 ± 0.07	1.11 ± 0.06	0.99
Glycine	0.71 ± 0.13	0.71 ± 0.14	0.76
Histidine	0.20 ± 0.06	0.19 ± 0.07	0.07
Hydroxyproline	−0.82 ± 0.16	−0.80 ± 0.17	0.19
Hypoxanthine	−1.50 ± 0.21	−1.50 ± 0.17	0.97
Isoleucine	0.01 ± 0.08	0.01 ± 0.08	0.11
L-Proline	0.40 ± 0.11	0.39 ± 0.11	0.23
Lactic acid	1.42 ± 0.13	1.41 ± 0.12	0.16
Leucine	0.29 ± 0.06	0.29 ± 0.06	0.91
Lysine	0.63 ± 0.07	0.63 ± 0.07	0.11
Malic acid	−0.91 ± 0.23	−0.90 ± 0.21	0.65
Methionine	−0.56 ± 0.11	−0.56 ± 0.11	0.98
*N*,*N*-Dimethylglycine	−1.13 ± 0.15	−1.11 ± 0.13	0.10
Ornithine	0.01 ± 0.09	0.01 ± 0.11	0.77
Phenylalanine	−0.03 ± 0.05	−0.02 ± 0.05	0.09
Pyruvic acid	0.40 ± 0.19	0.40 ± 0.15	0.87
Sarcosine	−1.38 ± 0.14	−1.37 ± 0.13	0.37
Serine	0.33 ± 0.11	0.32 ± 0.11	0.15
Threonine	0.37 ± 0.10	0.37 ± 0.09	0.32
Tryptophan	−0.06 ± 0.08	−0.07 ± 0.08	<0.05
Tyrosine	0.02 ± 0.07	0.03 ± 0.06	<0.05
Uridine	−0.67 ± 0.09	−0.66 ± 0.09	0.20
Valine	0.61 ± 0.05	0.60 ± 0.06	0.34

PINT, pain threshold of intraepidermal electrical stimulation.

### Correlation of the PINT index with clinical parameters, including the serum tryptophan level

Univariate regression analysis revealed a significant correlation between the PINT index and clinical measures, such as age, BMI, abdominal circumference, waist circumference, Fat, FBG, HbA1c, HOMA-IR, BUN, 8-OHdG, sBP, tryptophan, 2-hydroxy butyric acid, hypertension (+/−) and ATR (Table 4). Factors other than tryptophan have been shown to be related to pain thresholds in previous reports.^12-17^ The correlation between the PINT index and the tryptophan concentration remained significant after adjustment for age and sex (*β* = −0.08, *P* < 0.05; Table 5). The correlation between the PINT index and the tryptophan level (*β* = −0.09, *P* < 0.05) also remained significant after adjustment for multiple factors correlated with the PINT index in univariate analysis (age, BMI, waist circumference, Fat, HbA1c, BUN, 8-OHdG, sBP, 2-hydroxy butyric acid and ATR). These results also confirm that tryptophan metabolism can be a novel factor related to pain threshold and a novel candidate for a biomarker of SFN.

**Table 4:** Clinical factors correlated with PINT

	Univariate		Multivariate	
Characteristics	*β*	*P*	*β*	*P*
Sex	0.025755	0.4106		
Age (years)	0.114992	0.0002	0.008599	0.8575
Height (cm)	−0.03442	0.2714		
Body weight (kg)	0.058347	0.0621		
BMI (kg/m^2^)	0.095749	0.0022	0.077079	0.4350
Fat (%)	0.068052	0.0295	−0.08856	0.1624
Waist circumference (cm)	0.10186	0.0011	0.049353	0.5160
FBG (mg/dL)	0.107183	0.0006		
HbA1c (%)	0.104781	0.0008	0.064376	0.0690
F-IRI (μU/mL)	0.052502	0.0933		
HOMA-IR	0.065833	0.0353		
HOMA-β	−0.02295	0.4634		
BUN (mg/dL)	0.070049	0.0251	0.010375	0.7925
Cr (mg/dL)	0.028759	0.3581		
sBP (mmHg)	0.090759	0.0037	0.038604	0.3201
dBP (mmHg)	0.059311	0.0579		
Tc (mg/dL)	0.036056	0.2492		
Tg (mg/dL)	0.040784	0.1924		
HDL-c (mg/dL)	−0.03994	0.2018		
LDL-c (mg/dL)	0.040415	0.1965		
IL-6 (mg/dL)	0.025613	0.4132		
Hs-CRP (mg/dL)	0.045196	0.1486		
Pentosidine (pmol/mL)	0.034169	0.2749		
Urine 8-OHdG (ng/mg·Cr)	0.072521	0.0204	0.064376	0.0690
Hypertension (+/−)	0.082425	0.0084		
Dyslipidaemia (+/−)	−0.00223	0.9431		
Alcohol habit (+/−)	−0.00311	0.9210		
Smoking habit (+/−)	0.012851	0.6817		
Decreased ATR (+/−)	−0.08089	0.0160	−0.03491	0.3119
Subjective symptoms (+/−)	0.055039	0.0785		
Tryptophan	−0.10231	0.0010	−0.07615	0.0429
2-Hydroxy butyric acid	0.65107	0.0373	0.033478	0.3256

BMI, body mass index; FBG, fasting plasma glucose; F-IRI, fasting immunoreactive insulin; HOMA-β, homeostatic model assessment of β cell function; HOMA-IR, homeostatic model assessment of insulin resistance; sBP, systolic blood pressure; dBP, diastolic blood pressure; Tc, total cholesterol; Tg, triglyceride; HDL-c, high-density lipoprotein cholesterol; LDL-c, low-density lipoprotein cholesterol; IL-6, interleukin-6; Hs-CRP, high-sensitivity C-reactive protein; 8-OHdG, 8-hydroxy-2′-deoxyguanosine; ATR, Achilles tendon reflex; PINT, pain threshold of intraepidermal electrical stimulation.

**Table 5:** Correlation of age- and sex-adjusted tryptophan with PINT index

	Univariate		Age and gender adjusted		Multivariate	
	*β*	*P*	*β*	*P*	*β*	*P*
Tryptophan	−0.10231	0.0010	−0.07831	0.0235	−0.08735	0.0203

PINT, pain threshold of intraepidermal electrical stimulation.

## Discussion

We investigated the association between plasma metabolites evaluated by comprehensive metabolomic analysis and the pain threshold of the foot in this study. Although PCO analysis was not different between PINT-low and PINT-high participants, we first revealed a significant association between the tryptophan/propionate metabolic pathway and the PINT index, regardless of the presence of aberrant glycaemic metabolism. The standardized value of tryptophan and 2-hydroxybutyric was significantly decreased in PINT-High subjects compared to PINT-Low subjects, even after excluding patients with aberrant glycaemic metabolism. Univariate analysis revealed a significant correlation between a high PINT index and age, BMI, Fat, HbA1c, FBG, BUN, systolic blood glucose, 8-OHdG and tryptophan. The tryptophan level was the only correlated factor in multivariate analysis, even after adjustment for age and sex. These results suggest that the change in tryptophan metabolism is significantly correlated with an abnormal PINT index in the Japanese population.

Our report first revealed the relationship between pain threshold and changes in blood metabolomes in a large sample size of >1000 cases. To date, several reports have been published on metabolite changes in peripheral neuropathy by blood metabolome analysis.^23,26-30^ In posttraumatic and postoperative neuropathic pain, several metabolites involved in inflammatory processes, central nerve system functioning and neural signalling changed, but there was no correlation between pain intensity and the levels of metabolites.^29^ Regarding DPN in type 2 diabetes, the level of the serum metabolites phenylacetylglutamine, sorbitol, serine and dihydroceramide was associated with disease severity and pathogenesis.^23,26,30^ However, the presence of peripheral neuropathy does not have a great impact on metabolic pathways in the plasma of obese subjects.^27^ Even regarding DPN, the results have been inconsistent because the disease severity and duration and ethnicity may influence the results. Furthermore, the sample size was fewer than 100 cases in most reports, which may not be sufficient to obtain confident results. Thus, considering the sample size, our study is assumed to yield a certain level of convincing results with data reliability.

Tryptophan is one of nine essential amino acids in humans. It is an indispensable substance for maintaining human health that the human body cannot synthesize in sufficient amounts. Currently, supplements containing tryptophan are widely used for diseases such as insomnia, jet lag and depression. Dietary tryptophan is important as a precursor of hormones such as serotonin and melatonin and of biological pigments such as kynurenine and its metabolite nicotinamide adenine dinucleotide, which is an active substance of niacin.^41^ Tryptophan is degraded via four pathways^42^ (Supplementary Fig. 2). The major pathway is the kynurenine pathway by oxidation.^43^ The remaining minor pathways include the serotonin pathway by hydroxylation, the tryptamine pathway by decarboxylation and the indole pathway by transamination. However, the association between tryptamine and peripheral neuropathy has not been reported to date.

The present results showed that the tryptophan metabolic pathway and decreased blood tryptophan levels were involved in worsening of the pain threshold regardless of abnormalities in glucose metabolism. In the tryptophan metabolic pathway, only 1–4% of dietary tryptophan is degraded to serotonin, whereas >95% of tryptophan is metabolized by the tryptophan–kynurenine pathway (Supplementary Fig. 2), which synthesizes various physiologically active metabolites such as neuroprotective antioxidants, neuroprotective substances, toxic oxidants, neurotoxins, immunomodulators and pain.^43-46^ It may be difficult to determine which pathways related to tryptophan metabolism are responsible for suppressing pain sensation from the results of this study, because the serum levels of kynurenine and serotonin measured via metabolic analysis were too low for further evaluation. Therefore, further detailed measurements of the amounts of the metabolites involved in the tryptophan pathway are needed. Another possibility is that trivial changes in each metabolite, which were not detected as changes in individual metabolites, can result in entire change in the tryptophan metabolism pathway. Similarly, it seems to be difficult to determine whether the loss of neuroprotective effects or increased neurotoxic effects caused by changes in tryptophan metabolism would deteriorate pain thresholds. It will be useful to evaluate the pathological changes in small fibres to further understand the underlying mechanism in the future.^3,4,6^

The kynurenine pathway is the major route for tryptophan conversion in the brain and in the periphery.^43^ Peripheral concentration of kynurenine may directly affect the activity of the pathway within the brain because kynurenine can easily cross the blood–brain barrier.^47^ Kynurenine is assumed to be a neuroprotective metabolite because of its endogenous antioxidant nature.^48^ Furthermore, as of late, the kynurenine pathway has been known as a particular mechanism of chronic pain.^46, 49,50^ The kynurenine pathway is further divided into the neuroprotective kynurenic acid pathway and the neurotoxic quinolinic acid pathway.^51^ Kynurenic acid is an endogenous nonselective antagonist of all subtypes of excitatory amino acid receptors, such as glutamatergic receptors that take part in pain transmission.^52^ Kynurenic acid displays antioxidative properties.^53^ Kynurenic acid can suppress pain, neurogenic inflammation and cognitive dysfunction by acting on glutamate receptors.^54-59^ Because our previous report indicated that an increase in pain threshold is involved in the inflammatory factors, the anti-inflammatory effects of kynurenic acid can be decreased.^15^ Furthermore, decreased levels of kynurenic acid and increased levels of quinolinic acid are involved in the pathogenesis of both chronic pain and depression.^51,60^ These findings suggest that multiple trivial changes in tryptophan metabolic pathways may be involved in the change in pain sensation, even if each metabolite shows a lack of brisk changes.

In contrast, change in tryptophan metabolism can be induced by neurogenic inflammation.^61^ The activity of rate-limiting enzymes responsible for the first step of the tryptophan–kynurenine pathway is affected by proinflammatory factors (e.g. interferon-γ, lipopolysaccharide and tumour necrosis factor-α) *in vitro* and *in vivo*, resulting in an increase in the level of oxidative kynurenine metabolites such as 3-hydroxy-L-kynurenine, 3-hydroxyanthranilic acid and quinolinic acid.^62,63^ Thus, increased inflammation may reciprocally modulate tryptophan metabolism, resulting in deterioration of the pain threshold.

Quinolinic acid is a weak but specific agonist of NMDA receptor, a glutamatergic receptor that may cause excitotoxic neuronal cell loss.^43^ As an NMDA receptor agonist, quinolinic acid has been implicated in many neurological conditions, including inflammatory conditions.^64^ Increased NMDA receptor activity at the spinal cord plays a crucial role in the neuropathic pain caused by traumatic nerve injury and chemotherapy.^65,66^ Although these findings suggest that quinolinic acid may be involved in the modification of the pain threshold in our healthy subjects, further evaluation is needed to elucidate the implications of small fibre excitation in pain sensation.

A small amount of tryptophan is used for the synthesis of serotonin (1–4%^41^; Supplementary Fig. 2). As a neurotransmitter, serotonin participates in the regulation of pain, sleep, mood and so on. In the peripheral system, when tissue injury or inflammation occurs, serotonin is released from platelets and mast cells, relieving or aggravating pain.^67^ The pain process can be facilitated or inhibited depending on the specific subtypes and the distribution of serotonin receptors. The increased serotonin and noradrenaline innervation of the dorsal horn in streptozotocin (STZ) diabetic rats may account for enhanced pain during DPN.^68^ One of the major serotonin receptor subtypes expressed in rat dorsal root ganglion neurons is the 5-HT2A receptor. 5-HT2A receptors in the peripheral sensory terminals are responsible for serotonin-induced pain and hyperalgesia.^69^ Although tryptophan metabolism was correlated with a high PINT index in this study, the level of serotonin was too low and similar between PINT-low and PINT-high subjects. These results may suggest that the serotonin pathway is minimally involved in the high pain threshold.

The indole pathway is a metabolic pathway of tryptophan that leads to the synthesis of indole pyruvate by deamination. When gut bacteria process tryptophan, the resulting metabolites are indole, skatole, indole-3-acetic acid and indole-3-propionic acid (IPA),^70^ all of which may affect the host’s physiology.^71-73^ IPA is a metabolite produced exclusively by the gram-positive enterobacterium *Clostridium sporogenes* from dietary tryptophan. IPA accumulates in host serum and is increased by intermittent fasting. An increase in IPA levels in serum has been reported to enhance nerve regeneration in a mouse model of sciatic nerve crush.^74^ In STZ-induced diabetic rats, IPA treatment for 2 weeks markedly alleviated oxidative stress and endoplasmic reticulum stress in neuronal cells and attenuated pain behaviour.^75^ Thus, IPA has protective effects on neuronal injury. Interestingly, the present study found that propionic acid metabolism is also associated with a high PINT index, regardless of blood glucose level. Our previous report showed that a low abundance of *Bacteroides* in the gut was associated with a high pain threshold.^17^ Furthermore, changes in the *Bacteroides* population are reported to be correlated with indole propionic acid levels.^76^ These results indicate that the change in the indole pathway is possibly involved in the high pain threshold in healthy Japanese volunteers.

On the basis of our results, the tryptophan pathway might be clinically applied as a biomarker or a therapeutic target for the worsening of pain sensation. However, it is difficult to conclude that the results of the present study can be applied in therapeutic targeting of increased pain sensation because there is no information about the pathways or metabolites related to tryptophan metabolism that are involved in the modification of pain threshold. On the other hand, previous reports have shown that the kynurenine and tryptophan ratio, the kynurenic acid and quinolinic acid ratio and the serotonin and tryptophan ratio are correlated with chronic pain.^77-79^ Although the level of tryptophan was minimally reduced in our study, further changes could be seen by evaluating the metabolites-to-tryptophan ratio. These findings suggest that the ratio of tryptophan metabolites, rather than the level of sole tryptophan metabolites, can potentially be applied to predict the change in pain threshold associated with small fibre dysfunctions.

Our study has several limitations. First, this study is a population-based cross-sectional observation study. Because predesigned subjects with uniform backgrounds such as age, sex and clinical history were not used, it is unclear whether the development of SFN is directly associated with changes in blood metabolites. Whether the results of short-term or long-term changes in nutrients are associated with SFN was not revealed in this study. Thus, prospective designed longitudinal observation is required to validate our results in the future. Second, since invasive evaluation was not permitted in the Iwaki Study, no cutaneous or neural pathology or biochemical or molecular pathology evaluations were performed. To demonstrate whether the results of this study reveal the actual pathogenesis of SFN, it is necessary to verify the results by returning to animal studies or other methods in the future. However, it may be difficult to choose an appropriate model or stage for deteriorated pain sensation in healthy individuals in general. The mechanistic relationship between SFN and tryptophan metabolism needs to be examined at different stages of disease in several models. Third, this study clarified the correlation between the change in the serum metabolites and attenuation of the pain threshold. However, it is unclear whether these changes in metabolites can reflect the changes in the absolute level of metabolites. Addressing these drawbacks could lead to new diagnostic and therapeutic approaches for evaluating tryptophan metabolism in SFN.

Our current study first revealed that changes in tryptophan metabolism in the plasma were significantly associated with an elevated PINT index in >1000 subjects in the general Japanese population, and these associations may be independent of diabetic conditions. In addition to the possibility of a novel mechanism aggravating pain sensation, metabolites of the tryptophan metabolic pathway could be biomarkers or therapeutic targets for SFN. Nevertheless, it is still unclear whether these changes can be directly linked to the mechanistic and clinical manifestations of SFN. Further experimental confirmation with appropriate models is needed for future therapeutic and clinical applications.

## Supplementary Material

fcae103_Supplementary_Data

## Data Availability

Data cannot be shared publicly because of ethical concerns. Data are available from the Hirosaki University COI Institutional Data Access/Ethics Committee (contact via e-mail: coi@hirosaki-u.ac.jp) for researchers who meet the criteria for access to the data. Researchers need to be approved by the research ethics review board at the organization of their affiliation.
