# Supplementary material for: Tryptophan metabolism and small fibre neuropathy: a correlation study
Source: Brain Commun. 2024 Mar 25;6(2):fcae103. doi: 10.1093/braincomms/fcae103 (PMC11010654; doi:10.1093/braincomms/fcae103)
Supplement: fcae103_Supplementary_Data [file fcae103_supplementary_data.pdf]

Supplementary Table 1. Clinical profiles of the subjects divided by diabetic status

	nDM	IFG	DM
n (men: women)	887 (349:538)	51 (27:24)	83 (47:36)
Age (yrs)	52.87 ± 15.22	65.94 ± 10.30*	63.17 ± 12.51*
Height (cm)	161.23 ± 9.05	160.32 ± 8.39	161.12 ± 8.95
Body weight (kg)	59.24 ± 11.82	64.87 ± 14.67†	66.18 ± 12.62*
BMI (kg/m^2)	22.69 ± 3.47	24.10 ± 6.35†	25.46 ± 4.30*‡
Fat (%)	25.65 ± 7.92	28.90 ± 10.08†	29.10 ± 9.92†
Waist circumference (cm)	77.17 ± 10.11	85.51 ± 9.75*	86.25 ± 10.71*
FBG (mg/dL)	90.45 ± 8.25	116.49 ± 6.46*	129.53 ± 29.17*§
HbA1c (%)	5.55 ± 0.31	5.97 ± 0.28*	7.13 ± 1.29*§
F-IRI (μU/mL)	4.92 ± 2.68	8.07 ± 5.82*	10.26 ± 16.73*‡
HOMA-β	67.27 ± 36.83	54.46 ± 38.72$^{\#}$	42.08 ± 25.48*
HOMA-IR	1.12 ± 0.66	2.33 ± 1.72*	3.08 ± 3.85$^{*\,**}$
BUN (mg/dL)	14.33 ± 3.95	15.85 ± 3.82†	16.79 ± 5.21*
Cr (mg/dL)	0.71 ± 0.16	0.74 ± 0.16	0.90 ± 0.97*§
sBP (mmHg)	121.12 ± 17.30	136.86 ± 17.72*	136.28 ± 15.96*
dBP (mmHg)	70.81 ± 11.44	79.94 ± 11.85*	74.13 ± 10.40$^{\#\,**}$
Tc (mg/dL)	206.98 ± 34.19	210.39 ± 35.16	206.65 ± 33.94
Tg (mg/dL)	95.43 ± 68.51	116.29 ± 74.14$^{\#}$	124.42 ± 82.31†
HDL-c (mg/dL)	66.54± 17.22	62.14 ± 16.57	57.33 ± 16.23*
LDL-c (mg/dL)	115.26 ± 28.57	118.65 ± 29.5	118.41 ± 27.74
IL-6 (pg/mL)	1.50 ± 4.32	1.35 ± 1.08	1.79 ± 1.47
Hs CRP (mg/dL)	0.05 ± 0.08	0.09 ± 0.12†	0.11 ± 0.13*
Pentosidine (pmol/mL)	29.57 ± 15.66	32.98 ± 15.91	40.02 ± 41.89*‡
Urine 8-OHdG (ng/mg·Cr)	8.97 ± 3.97	9.52 ± 4.23	9.78 ± 6.35
Hypertension: n (%)	204/894 (25.22)	28/51 (54.90)*	51/83 (61.45)*
Dyslipidaemia: n (%)	102/894 (12.61)	9/51 (17.65)	31/83 (37.35)$^{*\,**}$
Alcohol habit: n (%)	415/894 (51.03)	30/51 (58.82)	42/83 (50.60)
Smoking habit: n (%)	139/894 (17.18)	3/51 (5.88)	15/83 (18.07)
Decreased ATR: n (%)	154/884 (17.42)	17/50 (34.00)†	24/83 (28.92)$^{\#}$
Subjective symptom: n (%)	15/889 (16.90)	2/51 (3.92)	2/83 (2.41)
PINT (mA)	0.15 ± 0.13	0.21 ± 0.21†	0.17 ± 0.15

nDM, nondiabetic subjects; IFG, impaired fasting glucose subjects; DM, type 2 diabetic subjects; BMI, body mass index; FBG, fasting plasma glucose; F-IRI, fasting immunoreactive insulin; HOMA-β, homeostatic model assessment of β cell function; HOMA-IR, homeostatic model assessment of insulin resistance; sBP, systolic blood pressure; dBP, diastolic blood pressure; Tc, total cholesterol; Tg, triglyceride; HDL-c, high-density lipoprotein cholesterol; LDL-c, low-density lipoprotein cholesterol; IL-6, interleukin-6. Hs-CRP, high sensitivity C-reactive protein; 8-OHdG, 8-hydroxy-2'-deoxyguanosine; ATR, Achilles tendon reflex; PINT, pain threshold of intraepidermal electrical stimulation. *p<0.001 vs. nDM, †p<0.01 vs. nDM, ‡p<0.001 vs. IFG, §p＜0.01 vs. IFG, $^{\#}$p<0.05 vs. nDM, **p<0.05 vs. IFG.

Supplementary Table 2. Clinical profiles of examined subjects divided by PINT index

	PINT-Low	PINT-High	p
N (men: women)	751 (307:444)	270 (116:154)	-
Age (yrs)	53.45 ± 15.17	56.88 ± 15.37	< 0.01
Height (cm)	161.32 ± 8.74	160.79 ±9.73	0.40
Body weight (kg)	59.51 ± 11.86	61.63 ± 13.05	< 0.05
BMI (kg/m^2)	22.72± 3.81	23.71 ± 3.73	< 0.001
Fat (%)	25.82 ± 8.17	26.82 ± 8.55	0.09
Waist circumference (cm)	77.61 ± 10.48	80.24 ± 10.56	< 0.001
FBG (mg/dL)	94.07 ± 16.11	97.27 ± 16.79	< 0.01
HbA1c (%)	5.66 ± 0.63	5.78 ± 0.67	< 0.01
F-IRI (μU/mL)	5.26± 5.30	6.21 ± 6.73	< 0.05
HOMA-β	64.70 ± 37.90	64.51 ± 33.80	0.94
HOMA-IR	1.26 ± 1.27	1.55 ± 1.79	< 0.01
BUN (mg/dL)	14.40 ± 4.19	15.19 ± 3.86	< 0.01
Cr (mg/dL)	0.72 ± 0.31	0.73 ± 0.33	0.62
sBP (mmHg)	122.34 ± 17.79	125.39 ± 18.28	< 0.05
dBP (mmHg)	71.14 ± 11.66	72.62 ± 11.25	0.07
Tc (mg/dL)	206.29 ± 33.31	209.41 ± 36.50	0.20
Tg (mg/dL)	97.46 ± 69.18	102.63 ± 74.00	0.30
HDL-c (mg/dL)	65.91± 17.28	64.64 ± 17.34	0.30
LDL-c(mg/dL)	114.85 ± 28.09	118.00 ± 29.68	0.12
IL-6 (pg/mL)	1.51 ± 4.34	1.53 ± 3.16	0.97
Hs CRP (mg/dL)	0.06 ± 0.09	0.07 ± 0.10	0.15
Pentosidine (pmol/mL)	30.55 ± 20.39	30.69 ± 16.18	0.92
Urine 8-OHdG (ng/mg·Cr)	8.90 ± 4.27	9.51 ± 4.07	< 0.05
Hypertension: n (%)	189/752 (25.13)	94/271 (34.69)	< 0.01
Dyslipidaemia: n (%)	97/752 (12.90)	45/271 (16.61)	0.15
Alcohol habit: n (%)	357/752 (47.47)	130/271 (47.97)	0.89
Smoking habit: n (%)	114/752 (15.16)	43/271 (15.87)	0.77
Decreased ATR: n (%)	138/749 (18.42)	57/268 (21.27)	0.32
Subjective symptom n (%)	12/752 (1.60)	7/270 (2.59)	0.30
PINT (mA)	0.09 ± 0.04	0.34 ± 0.15	< 0.0001

BMI, body mass index; FBG, fasting plasma glucose; F-IRI, fasting serum insulin; HOMA-β, homeostatic model assessment β cell function; HOMA-IR, homeostatic model assessment insulin resistance; sBP, systolic blood pressure; dBP, diastolic blood pressure; Tc, total cholesterol; Tg, triglyceride; HDL-c, high-density lipoprotein cholesterol; LDL-c, low-density lipoprotein cholesterol; IL-6, interleukin-6; Hs-CRP, high-sensitivity C-reactive protein; 8-OHdG, 8-hydroxy-2’-deoxyguanosine; ATR, Achilles tendon reflex; PINT, pain threshold of intraepidermal electrical stimulation.

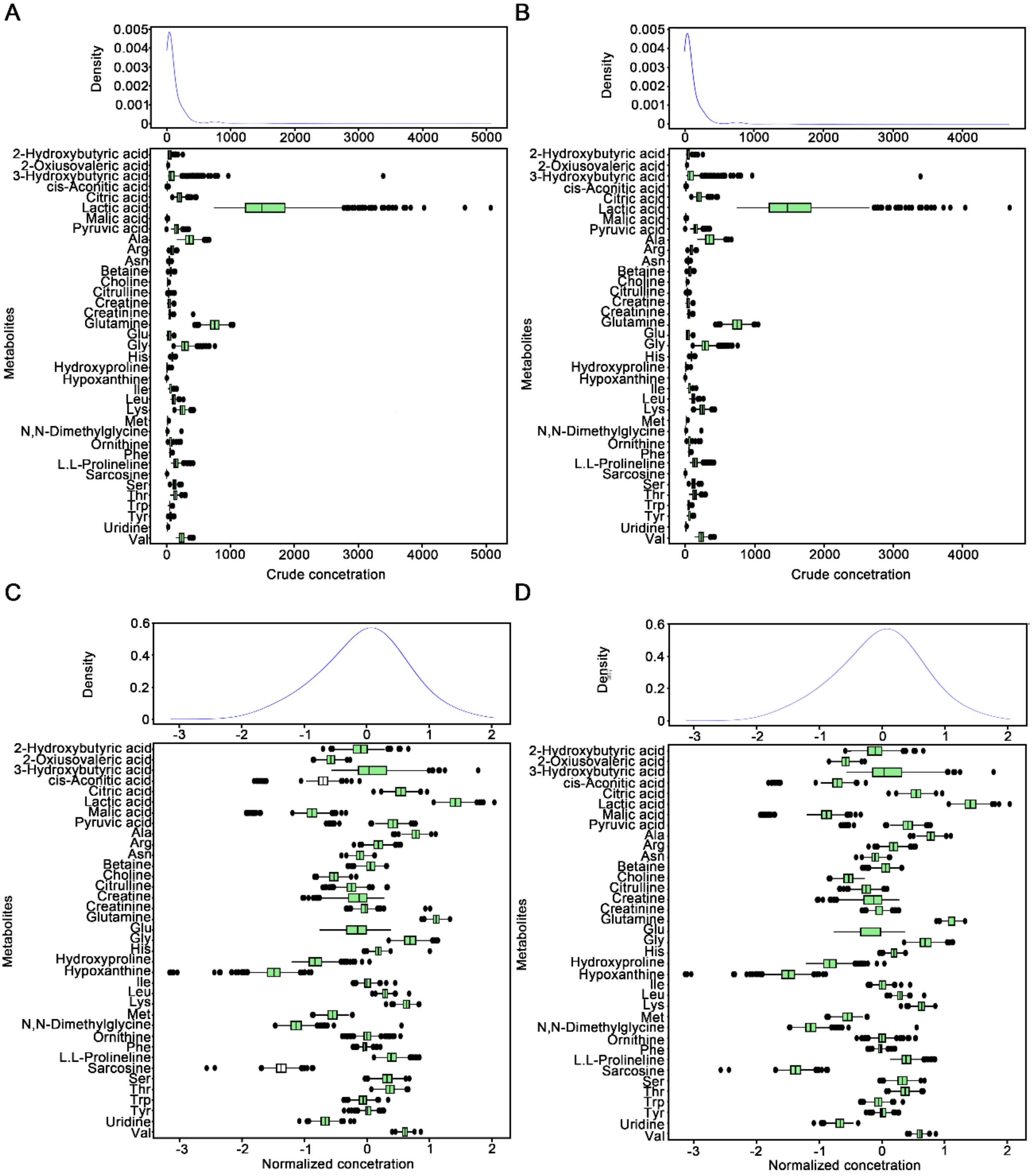

Supplementary Figure 1. Normalization of crude value of metabolites

The crude concentration of metabolites for whole subjects (**A**) and the subjects excluding abnormal glycemic state (**B**) was filtered and normalized using default settings in MetaboAnalystR (3.2.0). Density of mean normalized concentration was plotted around 0 in whole subjects (**C**) and the subjects excluding abnormal glycemic state (**D**).

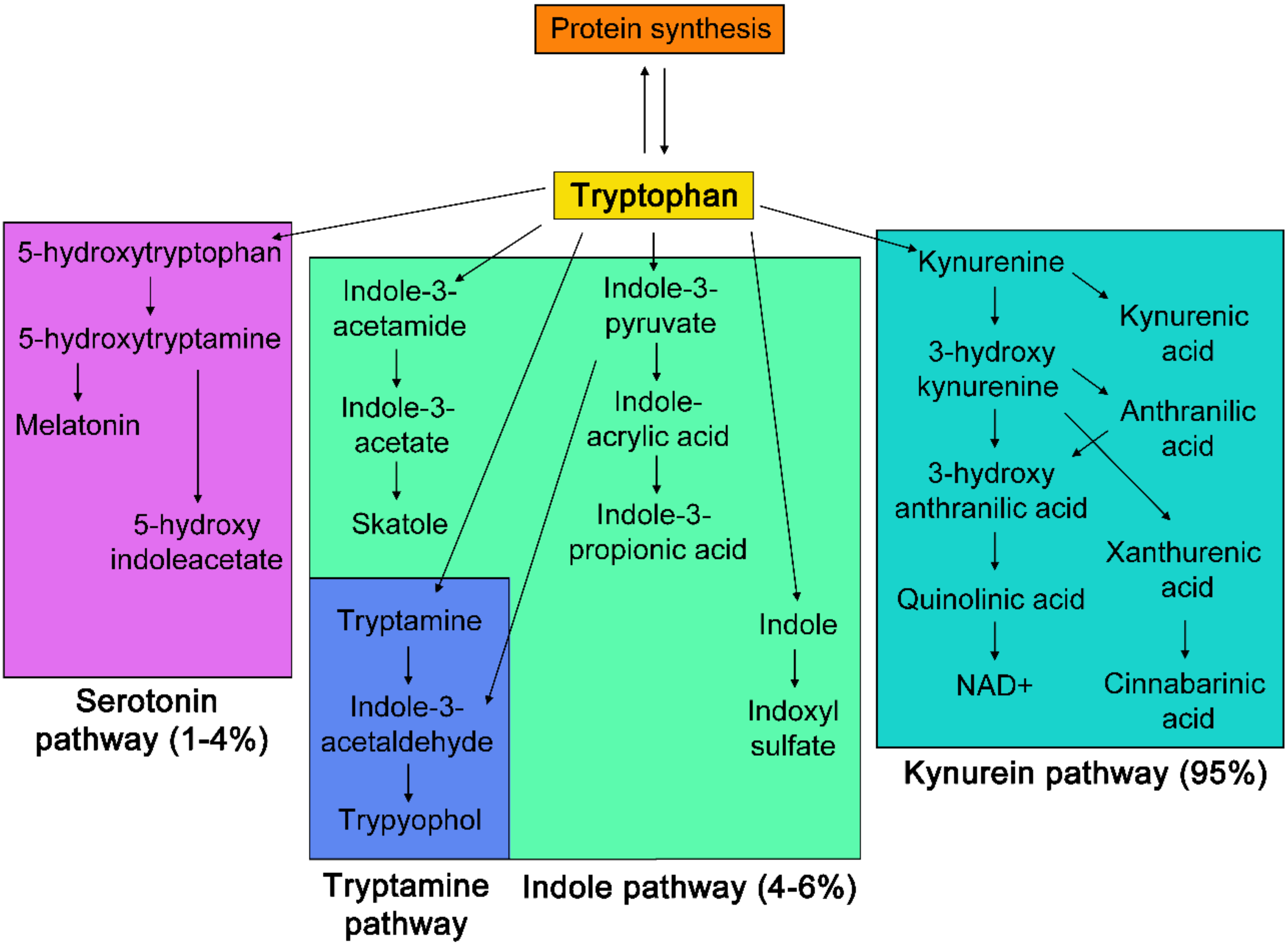

Supplementary Figure 2. Tryptophan metabolism

Tryptophan is degraded via four pathways including the kynurenine pathway, the serotonin pathway, the tryptamine pathway and the indole pathway. NAD, nicotinamide adenine dinucleotide. NAD+, nicotinamide adenine dinucleotide.
